# Effect of body mass and clothing on carrion entomofauna

**DOI:** 10.1007/s00414-015-1145-y

**Published:** 2015-01-27

**Authors:** Szymon Matuszewski, Katarzyna Frątczak, Szymon Konwerski, Daria Bajerlein, Krzysztof Szpila, Mateusz Jarmusz, Michał Szafałowicz, Andrzej Grzywacz, Anna Mądra

**Affiliations:** 1 Laboratory of Criminalistics, Adam Mickiewicz University, Św. Marcin 90, 61-809 Poznań, Poland; 2 Natural History Collections, Adam Mickiewicz University, Umultowska 89, 61-614 Poznań, Poland; 3 Department of Animal Taxonomy and Ecology, Adam Mickiewicz University, Umultowska 89, 61-614 Poznań, Poland; 4 Chair of Ecology and Biogeography, Nicolaus Copernicus University, Lwowska 1, 87-100 Toruń, Poland

**Keywords:** Forensic science, Forensic entomology, Postmortem interval, Carrion insects, Diptera, Coleoptera

## Abstract

Carcass mass largely affects pattern and rate of carrion decomposition. Supposedly, it is similarly important for carrion entomofauna; however, most of its likely effects have not been tested experimentally. Here, simultaneous effects of carcass mass and clothing are analyzed. A factorial block experiment with four levels of carcass mass (small carcasses 5–15 kg, medium carcasses 15.1–30 kg, medium/large carcasses 35–50 kg, large carcasses 55–70 kg) and two levels of carcass clothing (clothed and unclothed) was made in a grassland habitat of Western Poland. Pig carcasses (*N* = 24) were grouped into spring, early summer, and late summer blocks. Insects were sampled manually and with pitfall traps. Results demonstrate that insect assemblages are more complex, abundant, and long-lasting on larger carcasses, whereas clothing is of minor importance in this respect. Only large or medium/large carcasses were colonized by all guilds of carrion insects, while small or medium carcasses revealed high underrepresentation of late-colonizing insects (e.g., Cleridae or Nitidulidae). This finding indicates that carcasses weighing about 23 kg—a standard in forensic decomposition studies—give an incomplete picture of carrion entomofauna. Residencies of all forensically relevant insects were distinctly prolonged on larger carcasses, indicating that cadaver mass is a factor of great importance in this respect. The pre-appearance interval of most taxa was found to be unrelated to mass or clothing of a carcass. Moreover, current results suggest that rate of larval development is higher on smaller carcasses. In conclusion, this study demonstrates that carcass mass is a factor of crucial importance for carrion entomofauna, whereas the importance of clothing is small.

## Introduction

Carrion insects are useful for minimum postmortem interval (minimum PMI) estimation [1–3]. In forensic practice, minimum PMI is frequently determined from the age of immature insects or less frequently from the successional patterns of the carrion insect assemblages [2, 4]. The age of an insect may be estimated from the age indicators (e.g., developmental stage or larval length) by using temperature models for the development of particular species and case-specific temperature data [2, 5, 6]. This approach assumes that developmental rate is largely dependent on the temperature to which insects have been exposed, and this temperature largely depends on the ambient air temperature. Some other factors may be however also of importance, as for example the presence and size of larval masses [7–9]. The classical successional method involves analysis of the whole insect assemblage present on a cadaver, of which one chooses two “definitive taxa”: the one defining “lower PMI” and the other defining “upper PMI” [10–13]. Lower PMI relies on the pre-appearance interval (PAI) of the first definitive taxon, which may be estimated in many carrion insects from temperature [14–16] or may be determined by comparison with results of relevant pig carcass study [10, 11]. Upper PMI depends on the end of residency of the second definitive taxon (sometimes called “insect departure time”) [12, 13]. This moment in most carrion insects is only slightly related to preceding temperature [16]. Although seasonality affects to some extent the residency of carrion insects [17], currently, no single factor may be considered as a key factor in this respect. Therefore, in casework, the only way to determine the end of residency is a comparison with relevant pig carrion study [10, 11].

Several studies revealed a high importance of cadaver mass for pattern and rate of decomposition [18–23]. It seems that carcass mass may be similarly important for carrion entomofauna, and as such, it may confound development and succession-based estimates of minimum PMI. Surprisingly, its effects have so far only been tested in a single unreplicated experiment comparing carrion fauna of just two pig carcasses weighing 8.4 and 15.1 kg [18]. No size-related differences in composition of carrion fauna and patterns of its succession were reported, supposedly due to the small range of body mass included in the study [18]. This negative result should however not lead to a conclusion that cadaver mass is a factor of no or of small importance for insect fauna. Carrion entomofauna may be affected by carcass mass in several different ways. Firstly, body mass may structure composition of carrion insect assemblage. Carcasses of different mass represent different quantities and kind of resources available for insects. Larger carcasses have higher volume of muscles and higher fat content as compared to smaller carcasses [24, 25]. Moreover, active decay in larger carcasses is less efficient and much more biomass is left after its termination [23]. Insect assemblages on larger carcasses may therefore be more complex, with more larval taxa particularly of the late-colonizing species. Secondly, body mass may affect residency of carrion insects. It was demonstrated that bloating and active decay lasts longer on larger carcasses [23]. We hypothesize that residency of carrion insects may be similarly prolonged on larger carcasses. Thirdly, carcass mass may influence abundance of insects. Hewadikaram and Goff [18] observed a greater number of arthropods attracted to a larger pig. It seems that larger carcasses are colonized by greater number of larvae as well, which is suggested by greater number of larval masses on larger carcasses [23]. Moreover, it is reasonable to assume that larval masses are of larger volume on larger carcasses. Because the volume of the mass strongly influences its inner temperature [26], larvae may develop faster on larger bodies. Fourthly, carcass mass may affect the PAI of carrion insects. Larger carcasses lose heat at a slower rate [27] which may accelerate development of bacteria shortly after death and eventually may result in reduction of time needed for the production of insect attractants.

Carcass clothing is another factor of potential relevance for carrion entomofauna and entomological methods for minimum PMI estimation. Although its importance for pattern and rate of pig carrion decomposition is rather minor [23, 28, 29], it is believed that it may be very important for carrion insects [30]. Clothing affects abiotic conditions of the cadaver, as for example its humidity, amount of shade, or protection for inhabiting arthropods [30]. Some results indicate that it may increase abundance and diversity of carrion insects [30], enlarge dipteran larval masses and make larvae more freely moving [28–30], or prolong the period in which some larval Diptera are present on a cadaver [23, 29]. These effects were however not reported in every clothing-oriented study, so the importance of clothing for carrion entomofauna is still unclear.

In casework, cadavers differ according to their mass as well as kind and extent of clothing [23]. It is thus of vital importance for forensic practice to test whether these differences may confound PMI estimates from insect evidence. In this article, effects of carcass mass and clothing on entomofauna of pig carcasses are analyzed. We predict that (1) insect assemblages are more diverse and abundant on larger carcasses, (2) species residencies are longer on larger carcasses, (3) adult or larval PAIs are shorter on larger carcasses, (4) larval insects develop faster on large carcasses, (5) insect assemblages are more diverse on clothed carcasses, and (6) species residencies are longer on clothed carcasses.

## Materials and methods

### Experimental design

Experiment followed a complete factorial block design with carcass mass and carcass clothing as factors of interest. Four levels for carcass mass were studied (small carcasses 5–15 kg, medium carcasses 15.1–30 kg, medium/large carcasses 35–50 kg, and large carcasses 55–70 kg). Carcass clothing was considered on two levels (clothed and unclothed). Clothing consisted of trousers (with shortened legs), t-shirt, and a shirt or a blouse, all made of cotton and in similar colors. The whole sample (24 carcasses) was split into three blocks, with all levels of both factors studied in each block. The first block started in spring, the second in early summer, and the third in late summer. Carcasses were exposed, respectively, in the 17th of May, 16th of July, and 27th of August 2012. The average daily ground level temperatures ranged from 10.5 to 24.9 °C for the spring block, from 16.5 to 28.3 °C for the early summer block, and from 9.1 to 22.0 °C for the late summer block. Temperatures were logged hourly at every carcass by using HOBO U23 Pro v2 2x External Temperature Data Loggers (Onset Computer Corporation, MA, USA). The experiment followed a block design, so seasonal differences in temperature or insect abundance could have been isolated and treated as a separate “block” factor. Its effects on carrion entomofauna will not however be reported here, as they are neither novel nor interesting. A factorial block design was chosen for this experiment because we wanted to isolate and separate in this way temperature and seasonal effects on carrion insect fauna.

The study site was a xerothermic grassland situated in the Biedrusko military range (Western Poland, Europe; 52° 31′ N, 16° 55′ E). This is an area covered with grasses and no trees or large bushes. Eight sites along a roughly straight line were chosen in each block. Sites were indistinguishable according to abiotic and biotic conditions. The area was flat, in full sunlight, without any wind or sun barriers. Adjacent sites were at least 50 m apart.

### Carcasses

In total, 24 domestic pig carcasses (*Sus scrofa domestica* L.) were bought from a local commercial pig farm. Because we bought and used carcasses, there was no need to get the approval of the Ethics Commission on the Animal Experiments. Within each block, pigs were killed by a butcher at about 6 a.m. (a blow to the base of the skull) and after 1 to 3 h were exposed in the field. For the purpose of easy movement, carcasses were placed on a metal grate. In order to prevent scavenging, they were protected with welded wire mesh. Carcasses were weighed (a hanging scale KERN HCB 99 K50) just after exposure and then at varying intervals.

### Sampling and identification of insects

Samples were taken on a day-to-day basis until the 18th–20th day postmortem depending on the season. Afterwards, examinations were less frequent and intervals between subsequent samples increased with an increase in PMI. Insects were collected until no forensically relevant taxa were present. In the case of small or medium carcasses, it was about 50 days for the spring block, about 40 days for the early summer block, and about 80 days for the late summer block. In the case of medium/large and large carcasses, samples were taken until 19 November 2012. Because there was still much biomass on these carcasses and insects were still present, we decided to collect samples also in 2013 and 2014. However, sampling frequency and techniques in 2013 and 2014 were different compared to 2012. Moreover, only ten carcasses (five medium/large and five large) were included in this prolonged study. Accordingly, here we decided to report only results from 2012, as only samples collected in this year are suitable for comparing different classes of carcasses. Results from 2013 will be used only in analyses related to the composition of insect assemblages. Insect sampling was made always by two examiners and lasted usually 20–30 min per carcass (between 10 a.m. and 2 p.m.).

Two pitfall traps (plastic containers 16 cm in diameter and 17 cm in height) per carcass were used. Traps were filled with 50 % ethylene glycol solution and were emptied at every inspection. Manual collections consisted of aerial sweep net and hand samples and were made at every inspection. A swatting technique was performed twice (just two sweeps down onto the surface, one on the head, the other on the hind portion of a carcass) with large aerial sweep net (55 cm in diameter). Net samples were taken at the beginning of every inspection. Hand samples were taken using forceps from the carcass surface and from the soil under and near the carcass for about 10 min. Only these insects were sampled by hand which were unidentifiable in the field, and care was taken to sample only singular specimens from each taxon. Insects were killed and preserved in 70 % solution of ethanol. Care was taken to sample insects with the same intensity on all carcasses, so the intensity of sampling was not a confounding factor in this experiment.

Insect determinations were made using keys for identification [31–39]. Samples are deposited at the Laboratory of Criminalistics and Natural History Collections of Adam Mickiewicz University in Poznań, as well as at the Chair of Ecology and Biogeography of Nicolaus Copernicus University in Toruń.

### Data analyses

In order to analyze composition of insect assemblages, data from 2012 and 2013 were included. All the other analyses were made with data from 2012 only. While counting number of larval taxa per carcass, every instar from a given species was treated as a separate taxon regardless of its abundance and number of days on which it was recorded. When only third instar larvae of carrion breeding insects were collected or identified from a given carcass, it was assumed that first and second instars were also present on that carcass for some time prior to sampling of third instars.

Abundance was analyzed for most important species of Coleoptera (*Necrodes littoralis*, *Thanatophilus sinuatus*, *Thanatophilus rugosus*, *Creophilus maxillosus*, *Saprinus semistriatus*, *Saprinus planiusculus*, *Margarinotus brunneus*, *Dermestes frischii*, *Omosita colon*, *Nitidula rufipes*, *Necrobia violacea*, *Necrobia rufipes*) and Diptera (*Lucilia caesar*, *Lucilia sericata*, *Calliphora vomitoria*, *Phormia regina*, *Sarcophaga caerulescens*, *Stearibia nigriceps*, *Hydrotaea ignava*, *Hydrotaea pilipes*, *Fannia canicularis*, *Fannia leucosticta*). Only these species were included which were abundantly recorded in at least several samples and on at least several carcasses (Tables 1–2). Pooled pitfall trap and sweep net samples were included in these analyses. At first, the number of specimens of a given taxon collected on a given carcass was transformed into a portion of a total number of individuals from that taxon collected on all carcasses in a given block. Then these portions were averaged across all relevant taxa found on the carcass (separately in adult or larval Diptera or Coleoptera) and afterwards such generalized data were used in analyses.

**Table 1:** The occurrence of Diptera on different carcasses

Family	Species	Stage	Small carcasses		Medium carcasses		Medium/large carcasses		Large carcasses	
			C (*N* = 3)	U (*N* = 3)	C (*N* = 3)	U (*N* = 3)	C (*N* = 3)	U (*N* = 3)	C (*N* = 3)	U (*N* = 3)
Calliphoridae	*Calliphora vomitoria* (Linnaeus, 1758)	A	2	1	3	3	2	3	3	3
		L_3_	2	–	2	2	2	2	3	2
	*Lucilia caesar* (Linnaeus, 1758)	A	3	3	3	3	3	3	3	3
		L_3_	3	3	3	3	3	3	3	3
	*Lucilia sericata* (Meigen, 1826)	A	3	3	3	3	3	3	3	3
		L_3_	3	3	3	3	3	3	3	3
	*Phormia regina* (Meigen, 1826)	A	2	2	2	3	3	3	3	3
		L_3_	2	2	2	3	3	3	3	3
	*Protophormia terraenovae* (Robineau-Desvoidy, 1830)	A	–	1	–	1	3	2	2	1
		L_3_	–	1	–	1	2	1	2	1
Fanniidae	*Fannia canicularis* (Linnaeus, 1761)	A	2	2	2	2	1	3	3	3
		L_3_	2	2	1	1	–	2	2	2
	*Fannia leucosticta* (Meigen, 1838)	A	1	2	3	3	3	3	3	3
		L_3_	1	1	3	3	3	2	2	3
Muscidae	*Hydrotaea dentipes* (Fabricius, 1805)	A	1	1	2	2	2	2	3	3
		L_3_	1	1	2	1	2	1	3	1
	*Hydrotaea ignava* (Harris, 1780)	A	3	3	3	3	3	3	3	3
		L_3_	3	3	3	3	3	3	3	3
	*Hydrotaea pilipes* Stein, 1903	A	3	3	3	3	3	3	3	3
		L_3_	2	3	3	3	3	2	3	3
	*Hydrotaea similis* Meade, 1887	A	–	2	2	2	2	2	1	2
		L_3_	–	2	2	1	1	2	1	2
Piophilidae	*Stearibia nigriceps* (Meigen, 1826)	A	3	3	3	3	3	3	3	3
		L_3_	1	1	3	2	3	3	3	3
Sarcophagidae	*Sarcophaga caerulescens* (Zetterstedt, 1838)	A	2	3	2	3	3	3	2	3
		L_3_	2	3	2	3	3	3	2	3
	*Sarcophaga similis* Meade, 1876	A	3	2	1	2	1	2	3	2
		L_3_	3	2	1	2	1	2	3	1

Small carcasses 5–15 kg, medium carcasses 15.1–30 kg, medium/large carcasses 35–50 kg, large carcasses 55–70 kg

*C* clothed carcasses, *U* unclothed carcasses, *A* adult stage, *L*_*3*_ third instar larvae

**Table 2:** The occurrence of Coleoptera on different carcasses

Family	Taxon	Stage	Small carcasses		Medium carcasses		Medium/large carcasses		Large carcasses	
			C (*N* = 3)	U (*N* = 3)	C (*N* = 3)	U (*N* = 3)	C (*N* = 3)	U (*N* = 3)	C (*N* = 3)	U (*N* = 3)
Cleridae	*Necrobia rufipes* (DeGeer, 1775)	A	–	–	–	–	2	2	3	3
		L_1_	–	–	–	–	2	1	3	–
		L_2_	–	–	–	–	2	1	3	–
		L_3_	–	–	–	–	2	1	2	–
	*Necrobia violacea* (Linnaeus, 1758)	A	–	2	2	1	3	3	3	3
		L_1_	–	–	2	–	3	2	3	3
		L_2_	–	–	2	–	3	2	3	3
		L_3_	–	–	2	–	3	2	3	3
Dermestidae	*Dermestes frischii* Kugelann, 1792	A	2	2	2	2	3	3	3	3
		L_1_	1	2	1	2	3	3	3	3
		L_m_	1	2	1	1	3	3	3	2
Histeridae	*Carcinops pumilio* (Erichson, 1834)	A	–	–	–	–	1	–	1	1
	*Hister unicolor* Linnaeus, 1758	A	2	2	1	1	3	2	3	3
	*Margarinotus brunneus* (Fabricius, 1775)	A	3	3	3	3	3	3	3	3
	*Margarinotus carbonarius* (Hoffmann, 1803)	A	1	3	1	2	3	3	3	3
	*Margarinotus ventralis* (Marseul, 1854)	A	1	–	–	1	2	1	2	3
	*Margarinotus* sp.	L_1_	1	–	1	–	3	3	3	3
		L_2_	1	–	1	–	3	3	3	3
	*Saprinus planiusculus* Motschulsky, 1849	A	3	2	2	2	3	3	3	3
	*Saprinus semistriatus* (Scriba, 1790)	A	3	3	3	3	3	3	3	3
	*Saprinus aeneus* (Fabricius, 1775)	A	2	1	2	2	2	3	2	3
	*Saprinus* sp.	L_1_	2	2	2	2	2	2	2	1
		L_2_	2	2	2	2	2	2	2	1
Nitidulidae	*Omosita colon* (Linnaeus, 1758)	A	–	1	2	2	3	3	3	3
		L_1_	–	–	1	–	2	3	3	2
		L_2_	–	–	1	–	2	2	3	2
		L_3_	–	–	–	–	2	2	3	2
	*Nitidula rufipes* (Linnaeus, 1767)	A	–	–	2	–	2	3	3	3
	*Nitidula carnaria* (Schaller, 1783)	A	–	1	–	1	3	2	2	1
	*Nitidula* sp.	L	–	–	–	–	1	2	3	–
Silphidae	*Necrodes littoralis* (Linnaeus, 1758)	A	2	3	3	2	3	3	3	3
		L_1_	1	1	2	1	3	3	3	3
		L_2_	1	–	2	1	3	2	3	3
		L_3_	1	–	2	1	3	2	2	3
	*Thanatophilus rugosus* (Linnaeus, 1758)	A	3	3	3	3	3	3	3	3
	*Thanatophilus sinuatus* (Fabricius, 1775)	A	3	3	3	3	3	3	3	3
	*Thanatophilus* sp.	L_1_	2	2	2	3	3	3	3	3
		L_2_	2	2	2	3	3	3	3	3
		L_3_	2	2	2	2	3	3	3	3
Staphylinidae	*Creophilus maxillosus* (Linnaeus, 1758)	A	2	3	3	3	3	3	3	3
		L_1_	2	3	3	3	3	3	3	3
		L_2_	2	3	3	3	3	3	3	3
		L_3_	2	3	3	3	3	3	3	3
	*Philonthus* sp.	L_1_	–	–	2	1	3	3	3	3
		L_2_	–	–	1	1	3	3	3	3
		L_3_	1	2	3	2	3	3	3	3
Trogidae	*Trox sabulosus* (Linnaeus, 1758)	A	–	1	–	–	3	2	3	3

Small carcasses 5–15 kg, medium carcasses 15.1–30 kg, medium/large carcasses 35–50 kg, large carcasses 55–70 kg

*C* clothed carcasses, *U* unclothed carcasses, *A* adult stage, *L* larval stage, *L*_*1*_ first instar larvae, *L*_*2*_ second instar larvae, *L*_*3*_ third instar larvae, *L*_*m*_ mature larvae

Residency was defined as an interval from the first until the last presence of a taxon on a carcass. Residencies for higher taxa (adult or larval Diptera or Coleoptera) were calculated through averaging residencies across relevant lower taxa.

The PAI was defined as an interval from the moment of death until the first appearance of a given taxon. Analyses were made separately for adult and larval PAI.

ANOVA for block design was used in all analyses, with carcass mass, carcass clothing, and blocks as factors, and interaction between carcass mass and carcass clothing included. In all analyses, a 5 % level of significance was accepted. Calculations were made using Statistica 10 (StatSoft, Inc. 1984–2011).

## Results

### Composition and abundance of insects

**Fig. 1:**
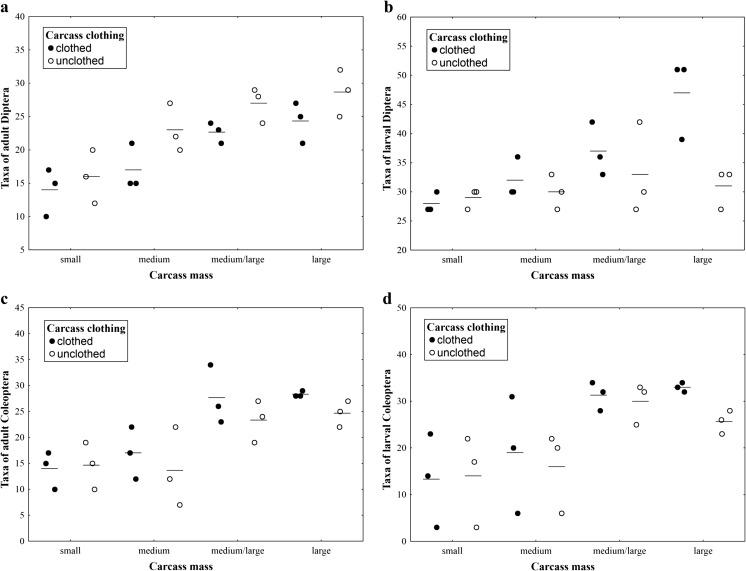
Effect of carcass mass and clothing on the number of taxa in adult or larval Diptera (**a** and **b**) or Coleoptera (**c** and **d**). Carcass mass: small 5–15 kg, medium 15.1–30 kg, medium/large: 35–50 kg, large: 55–70 kg. The *line* indicates the mean; the filled and empty circles indicate raw data

Carcass mass had a highly significant effect on the number of adult or larval taxa in both major carrion insect orders (adult Diptera, *F*_3,14_ = 20.5, *P* < 0.01; larval Diptera, *F*_3,14_ = 5.3, *P* = 0.01; adult Coleoptera, *F*_3,14_ = 14.5, *P* < 0.01; larval Coleoptera, *F*_3,14_ = 9.7, *P* < 0.01), with higher number of taxa on larger carcasses (Fig. 1). In particular, larger carcasses had higher number of late-arriving taxa, as for example beetles of the families Cleridae or Nitidulidae, which were collected on almost all medium/large and large carcasses, but were found on a very few small and medium pigs (Tables 1–2).

Carcass clothing revealed a significant effect on the number of taxa of adult or larval Diptera (adult Diptera, *F*_1,14_ = 13.3, *P* < 0.01; larval Diptera, *F*_1,14_ = 6.9, *P* = 0.02), a close-to-significant effect on the number of taxa of adult Coleoptera (*F*_1,14_ = 2.5, *P =* 0.14), and no significant effect on the number of taxa of larval Coleoptera (*F*_1,14_ = 1.0, *P =* 0.33). Clothed carcasses had more taxa of larval Diptera (Fig. 1b, Table 1), more taxa of adult or larval Coleoptera (Fig. 1c, d, Table 2), but less taxa of adult Diptera (Fig. 1a, Table 1). The interaction of carcass mass and clothing was significant only in the case of larval Diptera (*F*_3,14_ = 3.5, *P =* 0.45). Differences between clothed and unclothed carcasses enlarged with an increase in carcass mass (Fig. 1b).

**Fig. 2:**
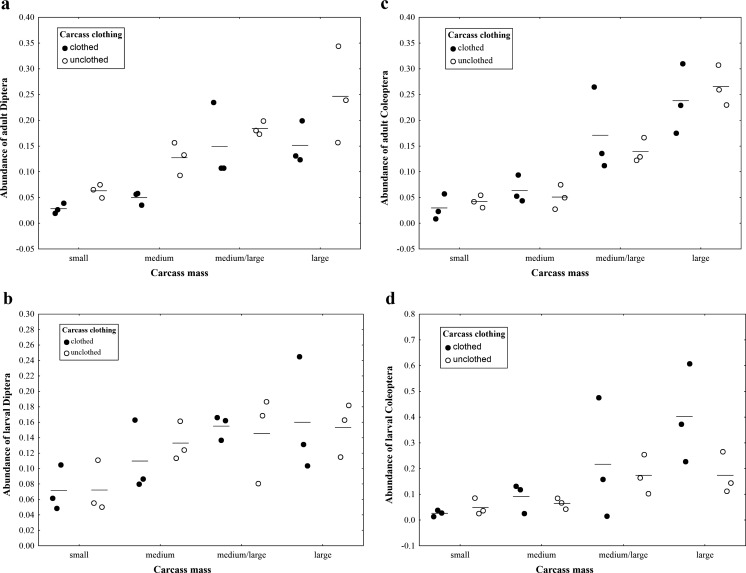
Effect of carcass mass and clothing on the abundance of insects averaged across the most important taxa of adult or larval Diptera (**a** and **b**) or Coleoptera (**c** and **d**). Number of individuals from a given taxon collected on a given carcass was expressed as a portion of a total number of individuals from that taxon collected on all carcasses in a given block. Carcass mass: small 5–15 kg, medium 15.1–30 kg, medium/large 35–50 kg, large 55–70 kg. The *line* indicates the mean; the filled and empty circles indicate raw data

Carcass mass showed a highly significant effect on the abundance of adult or larval Diptera or Coleoptera (adult Diptera, *F*_3,14_ = 11.8, *P* < 0.01; larval Diptera, *F*_3,14_ = 4.1, *P* = 0.03; adult Coleoptera, *F*_3,14_ = 26.7, *P* < 0.01; larval Coleoptera, *F*_3,14_ = 5.7, *P* < 0.01). Taxa from both orders had higher abundance on larger carcasses (Fig. 2).

Carcass clothing had a significant effect on the abundance of adult Diptera (*F*_1,14_ = 8.8, *P* = 0.01), which were more abundant on unclothed carcasses (Fig. 2a). There was also a close-to-significant effect of clothing on the abundance of larval Coleoptera (*F*_1,14_ = 2.1, *P =* 0.17; Fig. 2d) and no significant effect on the abundance of larval Diptera or adult Coleoptera (larval Diptera, *F*_1,14_ = 0.01, *P =* 0.92; Fig. 2b; adult Coleoptera, *F*_1,14_ < 0.01, *P =* 0.96; Fig. 2c). The interaction of carcass mass and clothing was insignificant in the case of all groups of taxa (*P* > 0.05).

### Residency and development of insects

No significant effect of carcass mass or clothing on the PAI was found in the majority of species (Table 3). As for the adult PAI, there was a significant effect of carcass mass just in the case of *C. maxillosus* and a near-significant effect in the case of *N. violacea* (Table 3). In both species, PAI was shorter on larger carcasses (Table 4). As for the larval PAI, there was a significant effect of carcass mass just in the case of *H. ignava* and a close-to-significant effect in the case of *L. caesar* (Table 3). Interestingly, both species revealed a shorter PAI on smaller carcasses (Table 4).

**Table 3:** Effect of carcass mass and clothing on the pre-appearance interval (PAI) of the most important adult and larval insects

Family	Species	Stage	Mass effect		Clothing effect	
			*F*	*P*	*F*	*P*
Silphidae	*Necrodes littoralis*	A	0.14	0.94	0.78	0.40
		L_1_	0.78	0.54	3.26	0.11
	*Thanatophilus sinuatus*	A	0.60	0.63	0.08	0.79
Staphylinidae	*Creophilus maxillosus*	A	4.24	0.03	1.30	0.27
		L_1_	1.17	0.38	0.70	0.42
Cleridae	*Necrobia violacea*	A	2.85	0.08	2.47	0.14
Dermestidae	*Dermestes frischii*	A	0.28	0.62	0.19	0.90
		L_1_	0.40	0.76	0.01	0.94
Histeridae	*Margarinotus brunneus*	A	0.95	0.45	0.36	0.56
	*Saprinus semistriatus*	A	0.91	0.46	0.02	0.90
Calliphoridae	*Lucilia caesar*	A	1.28	0.32	0.81	0.38
		L_3_	2.16	0.14	0.52	0.48
	*Lucilia sericata*	A	0.83	0.50	0.27	0.61
	*Phormia regina*	A	1.37	0.31	1.01	0.34
Muscidae	*Hydrotaea ignava*	A	1.95	0.18	4.00	0.07
		L_3_	8.40	<0.01	0.03	0.88
	*Hydrotaea pilipes*	A	0.23	0.87	0.01	0.94
Piophilidae	*Stearibia nigriceps*	A	1.44	0.28	0.93	0.35
		L_3_	0.49	0.70	0.06	0.82

*A* adult stage; *L*_*1*_ first instar larvae; *L*_*3*_ third instar larvae; *F* the value of *F* test (ANOVA); *P* the probability of *F*-statistic, assuming that there are no differences in PAI between different classes of carcasses

**Fig. 3:**
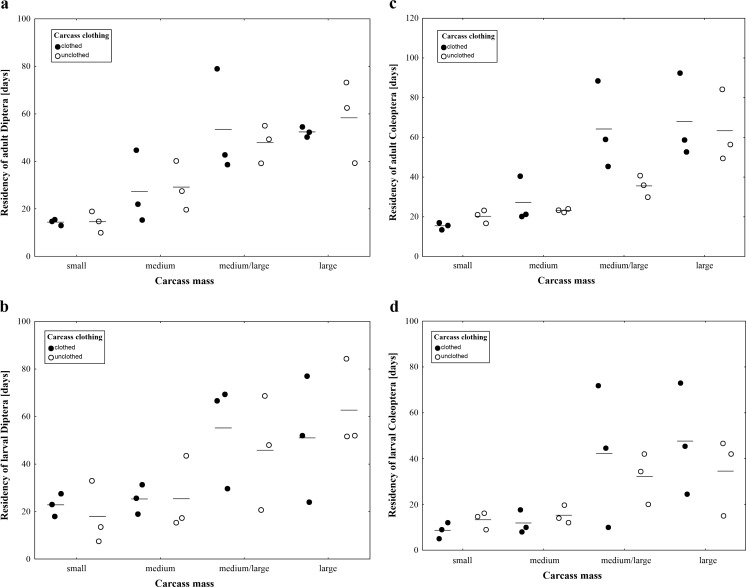
Effect of carcass mass and clothing on the residency of insects averaged across the most important taxa of adult or larval Diptera (**a** and **b**) or Coleoptera (**c** and **d**). Residency was defined as a period of a taxon presence on a carcass. Carcass mass: small 5–15 kg, medium 15.1–30 kg, medium/large 35–50 kg, large 55–70 kg. The *line* indicates the mean; the filled and empty circles indicate raw data

There was a highly significant effect of carcass mass on the residency of adult or larval Diptera or Coleoptera (adult Diptera, *F*_3,14_ = 21.5, *P* < 0.01; larval Diptera, *F*_3,14_ = 5.5, *P* = 0.01; adult Coleoptera, *F*_3,14_ = 30.2, *P* < 0.01; larval Coleoptera, *F*_3,14_ = 7.1, *P* < 0.01), with distinctly prolonged residencies on larger carcasses (Fig. 3).

Carcass clothing revealed a close-to-significant effect on the residency of adult Coleoptera (*F*_1,14_ = 4.1, *P* = 0.06), with longer residencies on clothed carcasses (Fig. 3c). Effect of clothing was insignificant in the other groups of taxa (adult Diptera, *F*_1,14_ = 0.02, *P =* 0.89, Fig. 3a; larval Diptera, *F*_1,14_ = 0.01, *P* = 0.93, Fig. 3b; larval Coleoptera, *F*_1,14_ = 0.42, *P* = 0.53; Fig. 3d). The interaction of carcass mass and clothing was insignificant in the case of all groups of taxa (*P* > 0.05).

**Fig. 4:**
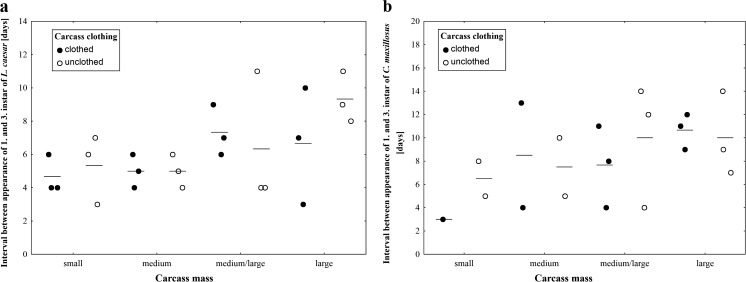
Effect of carcass mass and clothing on the interval between appearance of first and third instar larvae of *Lucilia caesar* (**a**) or *Creophilus maxillosus* (**b**). Carcass mass: small 5–15 kg, medium 15.1–30 kg, medium/large 35–50 kg, large 55–70 kg. The *line* indicates the mean; the filled and empty circles indicate raw data

Carcass mass revealed a near-significant effect on the interval between the appearance of first specimens of first instar larvae and first specimens of third instar larvae of *L. caesar* (*F*_3,9_ = 2.9, *P* = 0.07) and *C. maxillosus* (*F*_3,9_ = 1.8, *P* = 0.21). Interestingly, for both species, this interval was shorter on smaller carcasses (Fig. 4). The effect of clothing and the interaction of factors were not significant in the case of both species (*P* > 0.5).

**Table 4:** The average pre-appearance interval [days] of the most important adult and larval insects on carcasses of different mass

Family	Species	Stage	Small carcasses	Medium carcasses	Medium/large carcasses	Large carcasses
Silphidae	*Necrodes littoralis*	A	6.2	5.9	5.7	5.8
		L_1_	13.2	13.4	12.6	12.4
	*Thanatophilus sinuatus*	A	3.6	3.6	3.2	3.1
Staphylinidae	*Creophilus maxillosus*	A	6.8	7.2	6.4	4.6
		L_1_	12.6	12.5	15.7	14.7
Cleridae	*Necrobia violacea*	A	9.4	9.6	7.4	6.6
Dermestidae	*Dermestes frischii*	A	7.8	7.0	8.2	6.8
		L_1_	22.5	15.3	22.2	15.7
Histeridae	*Margarinotus brunneus*	A	6.6	6.9	6.2	5.1
	*Saprinus semistriatus*	A	5.1	5.3	5.4	4.1
Calliphoridae	*Lucilia caesar*	A	0.8	0.4	0.6	0.2
		L_3_	7.7	7.2	9.0	10.4
	*Lucilia sericata*	A	3.8	6.2	4.1	3.7
	*Phormia regina*	A	7.2	8.0	5.0	5.2
Muscidae	*Hydrotaea ignava*	A	5.2	5.7	9.7	7.1
		L_3_	8.1	9.7	12.3	14.1
	*Hydrotaea pilipes*	A	7.6	9.6	9.9	9.9
Piophilidae	*Stearibia nigriceps*	A	5.5	4.6	4.2	3.9
		L_3_	33.7	30.8	30.2	28.8

Small carcasses 5–15 kg, medium carcasses 15.1–30 kg, medium/large carcasses 35–50 kg, large carcasses 55–70 kg

*A* adult stage, *L*_*1*_ first instar larvae, *L*_*3*_ third instar larvae

## Discussion

The results presented here demonstrate that carrion entomofauna is more diverse on larger carcasses. A comparison with results of Hewadikaram and Goff [18] indicates that lack of differences in composition of carrion fauna between 8.4 and 15.1 kg carcasses, as recorded by these authors, must have resulted from small range of body masses included in their study. This interpretation is supported by our results, as differences between small and medium carcasses in the current experiment were relatively small.

It is of importance that increase of diversity on medium/large and large carcasses resulted from the regular presence of late-colonizing taxa, taxa which were usually absent on small carcasses or infrequent on medium carcasses. It is thus suggested that higher diversity of carrion entomofauna on larger carcasses comes down mainly to the presence of all guilds of carrion insects (i.e., early, middle, and late-arriving insects), not the presence of a higher number of species from each guild. These results substantiate the conclusion that only large carcasses (at least 35–50 kg) are colonized by all guilds of carrion insects. This finding of the current experiment has important implications for pig carcass studies in forensic entomology. Pig carcasses of 23 kg have been suggested for use in such studies [4]. Indeed, most researchers used carcasses of about 23 kg or smaller, as for example Perez et al. [40], Bygarski and LeBlanc [41], Prado e Castro et al. [42], or Benbow et al. [43] of the most recent, while larger carcasses were used very infrequently [44–47]. Current results however indicate that carcasses of 23 kg or smaller give an incomplete image of carrion entomofauna, with high underrepresentation of late-colonizing insects. Only medium/large or large carcasses had complete inventories of forensically relevant insects. Accordingly, based on the current results, we recommend larger carcasses (at least 35–40 kg) for use in forensically oriented pig carrion studies, particularly when first inventories or checklists of forensically relevant insects for a given area are to be made. Moreover, large carcasses may be necessary to study succession patterns of late-arriving insects, whereas small or medium carcasses may be considered as sufficient for studying early or middle-colonizing insects.

As for the effect of clothing, current results support the hypothesis that it enhances the diversity of carrion entomofauna. However, its effects are more subtle and clearly less uniform as compared to the effect of cadaver mass. These results are in line with results of previous studies [28, 29]. Surprisingly, the current study suggests that there are more taxa of adult Diptera on unclothed carcasses. Because adult Diptera were sampled mainly by means of aerial sweep net, we assume that this effect resulted from the way in which adult Diptera were collected. Clothing impedes collecting of flying insects, as they are often hidden under the clothing and, consequently, they cannot be sampled with a net. Moreover, our results suggest that carcass mass and clothing may interact, resulting in more profound effects of clothing on insect diversity in the case of larger carcasses. Such interaction was observed only in the case of larval Diptera, indicating that this may not be uniform across insect taxa.

As expected, abundance of insects increased with an increase of carcass mass, which is in line with results of Hewadikaram and Goff [18]. All these data support the hypothesis that carrion insect assemblages are more abundant on larger cadavers, although it should be noted that this effect results partly from longer residency of insects on larger carcasses and longer periods of insect sampling. Clothing is clearly less important in this respect.

Contrary to expectations, the pre-appearance interval (PAI) was unrelated to carcass mass in most taxa. It is also demonstrated that carcass clothing and PAI are not related to each other. These results are particularly important for forensic entomology, as they indicate that both factors of the current experiment may be considered as unimportant while estimating PAI in forensic practice. It was suggested that PAI of some forensically important insects may be estimated in a context-free way by using species-specific temperature models for PAI and case-specific temperature data [48, 49]. This view is supported by current results. Moreover, the present study indicates that temperature models for PAI may be created in experiments with medium carcasses, unless late-arriving insects are studied.

In taxa with significant effect of body mass on PAI, two different patterns were recorded. Some taxa revealed a decrease in PAI on larger carcasses, whereas some taxa showed an increase in PAI on such carcasses. The former pattern was actually the one which had been expected to be the case, although present results indicate that it is uncommon and practically unimportant. The latter pattern is difficult to explain. It was recorded only in the case of third instar larval PAI of *H. ignava* and *L. caesar*, and not in the case of conspecific adult PAI. Therefore, earlier appearance of third instar larvae on smaller carcasses was not a consequence of earlier arrival of conspecific adult insects, but probably shorter periods of first and second instar larval development. Perhaps, it may be explained in terms of higher rate of development on smaller carcasses.

Current results demonstrate that carcass mass is a factor of great importance for residency of carrion insects, whereas carcass clothing is rather unimportant in this respect. Residencies on large carcasses were regularly at least three times longer as compared to small carcasses. This finding has profound implications for all methods of PMI estimation which are based on insect residencies, as for example classical succession-based method [12, 13] or newer succession-based methods [50]. Current results show that all these methods are strongly mass-dependent. In particular, when the mass of a case-cadaver is higher than the mass of reference carcasses, the upper PMI will be systematically underestimated with the classical succession-based method. Because most of the baseline successional data were collected in experiments with carcasses weighing about 20 kg [17, 51, 52], and most cases involve cadavers of adults (50–90 kg), a residency-based methods will regularly underestimate upper PMI in forensic practice.

Surprisingly, *L. caesar* and *C. maxillosus* revealed decrease of an interval between the appearance of first and third instar larvae on smaller carcasses. This effect suggests that development time through the first and second larval instar is shorter on smaller carcasses. Smaller carcasses have smaller number of larval masses [23], usually one or two covering most of the body and being more compacted compared to masses on large carcasses, which are situated usually in separate areas of the body (Matuszewski S., personal observation). It is thus possible, that larval masses on smaller carcasses generate greater amounts of inner heat, resulting in increase of developmental rate. This pattern not necessarily has to be explained in terms of temperature effects on development, as recent study indicated that temperature is not the only important factor governing development in larval masses [9]. As for the *C. maxillosus*, it is one of the most forensically significant species of beetles [49, 53–56]. In the adult or larval stage it feeds on larval or pupal blowflies, usually resides in the ground under the body, and may be very abundant, particularly on large carcasses [49, 55, 56]. Although it does not form conspecific larval masses, its larvae may feed on masses of blowfly larvae and may eventually be exposed to the blowfly-generated heat. Moreover, development of larval insects (flies or beetles) may be accelerated on small carcasses due to shortage of food resources and resultant increased intra- or interspecific competition, as it was demonstrated that competition leads to an earlier emigration or pupariation of larval blowflies on carrion [57, 58].

Interestingly, effects of clothing were insignificant or of minor size in all analyses. These results suggest that importance of clothing is overrated in forensic entomology. It is however of importance that we used moderate clothing made just of cotton. Perhaps, if different types of clothing were used, i.e., heavier or made of synthetic fabric, effect of clothing would be larger.

## References

[1] Amendt J, Campobasso CP, Gaudry E, Reiter C, LeBlanc HN, Hall MJ (2007). Best practice in forensic entomology—standards and guidelines. Int J Legal Med.

[2] Wells JD, LaMotte NL, Byrd JH, Castner JL (2010). Estimating the postmortem interval. Forensic entomology: the utility of arthropods in legal investigations.

[3] Amendt J, Richards CS, Campobasso CP, Zehner R, Hall MJ (2011). Forensic entomology: applications and limitations. Forensic Sci Med Pathol.

[4] Catts EP, Goff ML (1992). Forensic entomology in criminal investigations. Annu Rev Entomol.

[5] Villet MH, Amendt J (2011) Advances in entomological methods for death time estimation. In: Turk EE (ed) Forensic pathology reviews, vol 6. Springer, pp 213–237

[6] Villet MH, Richards CS, Midgley JM, Amendt J, Campobasso CP, Goff ML, Grassberger M (2010). Contemporary precision, bias and accuracy of minimum post-mortem intervals estimated using development of carrion-feeding insects. Current concepts in forensic entomology.

[7] Slone DH, Gruner SV (2007). Thermoregulation in larval aggregations of carrion-feeding blow flies (Diptera: Calliphoridae). J Med Entomol.

[8] Charabidze D, Bourel B, Gosset D (2011). Larval-mass effect: characterisation of heat emission by necrophageous blowflies (Diptera: Calliphoridae) larval aggregates. Forensic Sci Int.

[9] Johnson AP, Wallman JF (2014). Effect of massing on larval growth rate. Forensic Sci Int.

[10] Goff ML, Omori AI, Gunatilake K (1988). Estimation of postmortem interval by arthropod succession. Three case studies from the Hawaiian Islands. Am J Forensic Med Pathol.

[11] Goff ML, Flynn MM (1991). Determination of postmortem interval by arthropod succession: a case study from the Hawaiian islands. J Forensic Sci.

[12] Schoenly K, Goff ML, Early M (1992). A basic algorithm for calculating the postmortem interval from arthropod successional data. J Forensic Sci.

[13] Schoenly K, Goff ML, Wells JD, Lord WD (1996). Quantifying statistical uncertainty in succession-based entomological estimates of the postmortem interval in death scene investigations: a simulation study. Am Entomol.

[14] Matuszewski S, Szafałowicz M (2013). Temperature-dependent appearance of forensically useful beetles on carcasses. Forensic Sci Int.

[15] Matuszewski S, Szafałowicz M, Grzywacz A (2014). Temperature-dependent appearance of forensically useful flies on carcasses. Int J Legal Med.

[16] Archer M (2014). Comparative analysis of insect succession data from Victoria (Australia) using summary statistics versus preceding mean ambient temperature models. J Forensic Sci.

[17] Matuszewski S, Bajerlein D, Konwerski S, Szpila K (2011). Insect succession and carrion decomposition in selected forests of Central Europe. Part 3: succession of carrion fauna. Forensic Sci Int.

[18] Hewadikaram KA, Goff ML (1991). Effect of carcass size on rate of decomposition and arthropod succession patterns. Am J Forensic Med Pathol.

[19] Komar D, Beattie O (1998). Effects of carcass size on decay rates of shade and sun exposed carrion. Can Soc Forensic Sci J.

[20] Simmons T, Adlam RE, Moffatt C (2010). Debugging decomposition data—comparative taphonomic studies and the influence of insects and carcass size on decomposition rate. J Forensic Sci.

[21] Spicka A, Johnson R, Bushing J, Higley LG, Carter DO (2011). Carcass mass can influence rate of decomposition and release of ninhydrin-reactive nitrogen into gravesoil. Forensic Sci Int.

[22] Sutherland A, Myburgh J, Steyn M, Becker PJ (2013). The effect of body size on the rate of decomposition in a temperate region of South Africa. Forensic Sci Int.

[23] Matuszewski S, Konwerski S, Frątczak K, Szafałowicz M (2014). Effect of body mass and clothing on decomposition of pig carcasses. Int J Legal Med.

[24] Mitchell AD, Conway JM, Potts WJE (1996). Body composition analysis of pigs by dual-energy X-ray absorptiometry. J Anim Sci.

[25] Bruns KW, Pritchard RH, Boggs DL (2004). The relationships among body weight, body composition, and intramuscular fat content in steers. J Anim Sci.

[26] Slone DH, Gruner SV (2007). Thermoregulation in larval aggregations of carrion-feeding blow flies (Diptera: Calliphoridae). J Med Entomol.

[27] Madea B, Moenssens A, Jamieson A (2009). Death: time of. Wiley encyclopedia of forensic science.

[28] Kelly JA, van der Linde TC, Anderson GS (2009). The influence of clothing and wrapping on carcass decomposition and arthropod succession during the warmer seasons in central South Africa*. J Forensic Sci.

[29] Voss SC, Cook DF, Dadour IR (2011). Decomposition and insect succession of clothed and unclothed carcasses in Western Australia. Forensic Sci Int.

[30] Anderson GS, Byrd JH, Castner JL (2010). Factors that influence insect succession on carrion. Forensic entomology: the utility of arthropods in legal investigations.

[31] Szpila K, Amendt JC CP, Goff ML, Grassberger M (2010). Key for the identification of third instars of European blowflies (Diptera: Calliphoridae) of forensic importance. Current concepts in forensic entomology.

[32] Szpila K, Gennard D (2012). Key for identification of European and Mediterranean blowflies (Diptera, Calliphoridae) of medical and veterinary importance—adult flies. Forensic entomology, an introduction.

[33] Gregor F, Rozkošny R, Barták M, Vaňhara J (2002). The Muscidae (Diptera) of Central Europe.

[34] McAlpine JF (1977). A revised classification of the Piophilidae including ‘Neotophilidae’ and ‘Tyreophoridae’ (Diptera: Schizophora). Mem Entomol Soc Can.

[35] Keys for the identification of Polish insects, (1955–2008). Polish Entomological Society

[36] Freude H, Harde KW, Lohse GA (1971–1992) Die Käfer Mitteleuropas. Goecke & Evers, Krefeld

[37] Klausnitzer B (1991–2001) Die Larven der Käfer Mitteleuropas. Spektrum Akademisher Verlag, Heidelberg, Berlin

[38] Grzywacz A, Lindstrom A, Hall MJ (2014). Hydrotaea similis Meade (Diptera: Muscidae) newly reported from a human cadaver: a case report and larval morphology. Forensic Sci Int.

[39] Rozkošny R, Gregor F, Pont AC (1997). The European Fanniidae (Diptera). Acta Sci Nat Acad Sci Bohemicae Brno.

[40] Perez AE, Haskell NH, Wells JD (2014). Evaluating the utility of hexapod species for calculating a confidence interval about a succession based postmortem interval estimate. Forensic Sci Int.

[41] Bygarski K, LeBlanc HN (2013). Decomposition and arthropod succession in Whitehorse, Yukon Territory, Canada. J Forensic Sci.

[42] Prado e Castro C, García MD, Martins da Silva P, Faria e Silva I, Serrano A (2013). Coleoptera of forensic interest: a study of seasonal community composition and succession in Lisbon, Portugal. Forensic Sci Int.

[43] Benbow ME, Lewis AJ, Tomberlin JK, Pechal JL (2013). Seasonal necrophagous insect community assembly during vertebrate carrion decomposition. J Med Entomol.

[44] Wang J, Li Z, Chen Y, Chen Q, Yin X (2008). The succession and development of insects on pig carcasses and their significances in estimating PMI in south China. Forensic Sci Int.

[45] Sharanowski BJ, Walker EG, Anderson GS (2008). Insect succession and decomposition patterns on shaded and sunlit carrion in Saskatchewan in three different seasons. Forensic Sci Int.

[46] Özdemir S, Sert O (2009). Determination of Coleoptera fauna on carcasses in Ankara province, Turkey. Forensic Sci Int.

[47] Anderson GS (2011). Comparison of decomposition rates and faunal colonization of carrion in indoor and outdoor environments. J Forensic Sci.

[48] Matuszewski S (2011). Estimating the pre-appearance interval from temperature in *Necrodes littoralis* L. (Coleoptera: Silphidae). Forensic Sci Int.

[49] Matuszewski S (2012). Estimating the preappearance interval from temperature in *Creophilus maxillosus* L. (Coleoptera: Staphylinidae). J Forensic Sci.

[50] Michaud JP, Moreau G (2009). Predicting the visitation of carcasses by carrion-related insects under different rates of degree-day accumulation. Forensic Sci Int.

[51] Eberhardt TL, Elliot DA (2008). A preliminary investigation of insect colonisation and succession on remains in New Zealand. Forensic Sci Int.

[52] Anton E, Niederegger S, Beutel RG (2011). Beetles and flies collected on pig carrion in an experimental setting in Thuringia and their forensic implications. Med Vet Entomol.

[53] Dekeirsschieter J, Frederick C, Verheggen FJ, Drugmand D, Haubruge E (2013). Diversity of forensic rive beetles (Coleoptera, Staphylinidae) associated with decaying pig carcass in a forest biotope. J Forensic Sci.

[54] Mądra A, Konwerski S, Matuszewski S (2014). Necrophilous Staphylininae (Coleoptera: Staphylinidae) as indicators of season of death and corpse relocation. Forensic Sci Int.

[55] Frątczak K, Matuszewski S (2014). Instar determination in forensically useful beetles *Necrodes littoralis* (Silphidae) and *Creophilus maxillosus* (Staphylinidae). Forensic Sci Int.

[56] Watson–Horzelski EJ (2012). Survival and time of development for *Creophilus maxillosus* (L.) (Coleoptera: Staphylinidae) at three constant temperatures. Coleopt Bull.

[57] Ireland S, Turner B (2005). The effects of larval crowding and food type on the size and development of the blowfly, *Calliphora vomitoria*. Forensic Sci Int.

[58] Shiao AF, Yeh TC (2008). Larval competition of *Chrysomya megacephala* and *Chrysomya rufifacies* (Diptera: Calliphoridae): behavior and ecological studies of two blow fly species of forensic significance. J Med Entomol.

